# A Metabolic Obesity Profile Is Associated With Decreased Gray Matter Volume in Cognitively Healthy Older Adults

**DOI:** 10.3389/fnagi.2019.00202

**Published:** 2019-08-02

**Authors:** Frauke Beyer, Shahrzad Kharabian Masouleh, Jürgen Kratzsch, Matthias L. Schroeter, Susanne Röhr, Steffi G. Riedel-Heller, Arno Villringer, A. Veronica Witte

**Affiliations:** ^1^Department of Neurology, Max Planck Institute for Human Cognitive and Brain Sciences, Leipzig, Germany; ^2^Subproject A1, CRC 1052 “Obesity Mechanisms”, University of Leipzig, Leipzig, Germany; ^3^Institute of Neuroscience and Medicine (INM-7: Brain and Behaviour), Research Centre Jülich, Jülich, Germany; ^4^Institute of Laboratory Medicine, Clinical Chemistry and Molecular Diagnostics, University of Leipzig, Leipzig, Germany; ^5^Clinic for Cognitive Neurology, University of Leipzig, Leipzig, Germany; ^6^Institute of Social Medicine, Occupational Health and Public Health (ISAP), University of Leipzig, Leipzig, Germany

**Keywords:** obesity, leptin – adiponectin, aging, metabolic risk, multivariate analysis, VBM

## Abstract

Obesity is a risk factor for cognitive decline and gray matter volume loss in aging. Studies have shown that different metabolic factors, e.g., dysregulated glucose metabolism and systemic inflammation, might mediate this association. Yet, even though these risk factors tend to co-occur, they have mostly been investigated separately, making it difficult to establish their joint contribution to gray matter volume structure in aging. Here, we therefore aimed to determine a metabolic profile of obesity that takes into account different anthropometric and metabolic measures to explain differences in gray matter volume in aging. We included 748 elderly, cognitively healthy participants (age range: 60 – 79 years, BMI range: 17 – 42 kg/m^2^) of the LIFE-Adult Study. All participants had complete information on body mass index, waist-to-hip ratio, glycated hemoglobin, total blood cholesterol, high-density lipoprotein, interleukin-6, C-reactive protein, adiponectin and leptin. Voxelwise gray matter volume was extracted from T1-weighted images acquired on a 3T Siemens MRI scanner. We used partial least squares correlation to extract latent variables with maximal covariance between anthropometric, metabolic and gray matter volume and applied permutation/bootstrapping and cross-validation to test significance and reliability of the result. We further explored the association of the latent variables with cognitive performance. Permutation tests and cross-validation indicated that the first pair of latent variables was significant and reliable. The metabolic profile was driven by negative contributions from body mass index, waist-to-hip ratio, glycated hemoglobin, C-reactive protein and leptin and a positive contribution from adiponectin. It positively covaried with gray matter volume in temporal, frontal and occipital lobe as well as subcortical regions and cerebellum. This result shows that a metabolic profile characterized by high body fat, visceral adiposity and systemic inflammation is associated with reduced gray matter volume and potentially reduced executive function in older adults. We observed the highest contributions for body weight and fat mass, which indicates that factors underlying sustained energy imbalance, like sedentary lifestyle or intake of energy-dense food, might be important determinants of gray matter structure in aging.

## Introduction

Obesity is associated with adverse health consequences (World Health Organization [WHO], 2000). In particular, several studies have suggested that higher body mass index (BMI) in mid- and late life is associated with impairment in cognitive function (Debette et al., 2011; Smith et al., 2011; Singh-Manoux et al., 2012) and leads to a higher risk for dementia (Baumgart et al., 2015; Emmerzaal et al., 2015).

Potential mediators, among others, include metabolic risk factors, e.g., dysregulated glucose metabolism and chronic inflammation (Shaw et al., 2017; Corlier et al., 2018; Warren et al., 2018). Yet, these factors often co-occur and their individual role is difficult to establish. Consequently, the neurobiological mechanisms that link obesity and higher risk for cognitive decline in aging remain poorly understood.

Recent neuroimaging studies have provided neurobiological evidence of an association between the most common anthropometric measure of obesity, BMI, and decreased gray matter volume (GMV) (Gustafson et al., 2004; Enzinger et al., 2005; Taki et al., 2012; Bobb et al., 2014; Debette et al., 2014; Kharabian Masouleh et al., 2016).

Further studies have shown that waist-to-hip ratio (WHR), an indicator of visceral adiposity, might be a better predictor of GMV loss compared to BMI (Debette et al., 2010; Debette et al., 2014; Janowitz et al., 2015). This finding is in analogy with the increased cardiovascular risk associated with visceral fat accumulation (Lee et al., 2008).

Visceral adiposity often goes along with dyslipidemia, e.g., increased levels of triglycerides and low-density lipoproteins along with reduced levels of high-density lipoproteins (Klop et al., 2013). While dyslipidemia increases the risk for cardiovascular disease, its association with brain structure is still unclear (Assessment, 2009). Some studies showed that higher levels of total cholesterol and lower levels of high-density lipoprotein are associated with reduced GMV or cortical thickness (Ward et al., 2010; Walhovd et al., 2014), yet other studies have failed to replicate these findings (Leritz et al., 2011; Cox et al., 2019).

A vast amount of literature suggests that disturbances in glucose metabolism, ranging from hyperglycemia and insulin resistance to manifest diabetes, are associated with decreased GMV in middle-aged and older adults (Benedict et al., 2012; Kerti et al., 2013; Moran et al., 2013; Biessels and Reijmer, 2014; Reitz et al., 2016; Shaw et al., 2017; Repple et al., 2018). Insulin resistance might be one mediator of this association given the role of insulin in memory facilitation and regulation of amyloid-β (Craft, 2005; Blázquez et al., 2014; Cheke et al., 2017) in the brain. Accordingly, several studies have reported lower GMV in key memory regions like hippocampus and temporal lobe related to disturbances in glucose regulation (Benedict et al., 2012; Cherbuin et al., 2012; Kerti et al., 2013).

Systemic inflammation is another important metabolic factor with potential implications for brain health. Visceral adipose tissue secrets inflammatory cytokines which have been shown to impair the blood-brain-barrier and might thereby promote neuro-inflammation (Yaffe et al., 2004; Hsuchou et al., 2012). Accordingly, previous neuroimaging studies showed that circulating levels of pro-inflammatory cytokines such as C-reactive protein (CRP) and interleukin-6 (IL6) predict gray matter volume decline (Marsland et al., 2008; Papenberg et al., 2016; Corlier et al., 2018).

Other obesity-related metabolic factors that might have direct or indirect effects on brain function are adipose-tissue derived signaling hormones like leptin and adiponectin.

Leptin has multiple effects in the brain beyond its known role in the hypothalamic control of food intake. For instance, leptin signaling in the hippocampus plays an important role for memory (Paz-Filho et al., 2010; Irving and Harvey, 2013). First evidence from neuroimaging indicated that higher leptin levels might be neuroprotective and help to maintain memory function in older adults, mediated by hippocampus structure (Lieb et al., 2009; Narita et al., 2009; Witte et al., 2016). However, in obesity, leptin is often chronically elevated resulting in central resistance to the effects of the molecule (Myers et al., 2008).

Adiponectin is an adipokine originally known for its insulin-sensitizing and anti-inflammatory properties in the periphery (Lihn et al., 2005). Moreover, it was also suggested to exert beneficial effects on brain function, e.g., by modulating glucose metabolism (Cisternas et al., 2018) but to date, neuroimaging studies have not shown a consistent association of adiponectin and GMV (García-Casares et al., 2016; Hayakawa et al., 2018).

Taken together, different mechanisms might link obesity and related metabolic disturbances with brain health and cognitive function in aging. Most studies so far have focused on single, mostly anthropometric measures of obesity without taking into account related metabolic factors. Here, we use a multivariate method, called partial least squares correlation (PLSC) to derive informative patterns of covariation between anthropometric (overall and visceral adiposity) and metabolic measures (markers of energy metabolism, systemic inflammation and adipose-tissue derived hormones) of obesity and GMV in a sample of cognitively healthy older adults (McIntosh et al., 1996). PLSC is well-suited for data sets with highly correlated variables (e.g., neuroimaging data) and allows to jointly model behavioral and neuroimaging data. In particular, we chose PLSC over other multivariate methods such as canonical correlation analysis as it performs better in terms of predictive power when a high number of voxel are investigated (Grellmann et al., 2015).

We hypothesized a metabolic profile, which highlights detrimental aspects of obesity-related metabolic dysregulation to be associated with a pattern of GMV loss including medio-temporal areas (Cherbuin et al., 2012; Cheke et al., 2017). Furthermore, we aimed to explore the association of this profile with cognitive function.

## Materials and Methods

### Sample Selection

The study sample was selected from the LIFE-Adult study (Loeffler et al., 2015). The study was carried out in accordance with the Declaration of Helsinki and approved by the institutional ethics board of the Medical Faculty of the University of Leipzig. All subjects gave written informed consent.

We included 1222 older participants (≥60 years) with head magnetic resonance imaging (MRI) and without stroke, major brain pathology, cancer in the last 12 months or intake of centrally active medication. Out of these, we selected all participants with complete anthropometric and blood plasma measurements.

We measured body weight, height, waist and hip circumference with a precision of 0.01 kg and 0.1 cm, respectively, and calculated BMI and WHR.

Markers of long-term glucose metabolism (HbA1c), lipid metabolism (total cholesterol and high-density lipoprotein, HDL), systemic inflammation (CRP and IL6) were obtained after overnight fasting according to standard procedures (Loeffler et al., 2015).

Immunoreactive leptin and adiponectin concentrations were measured from fasted serum samples using immunoreactive kits (sensitive ELISA, Mediagnost, Reutlingen).

We excluded participants who scored below 27 in the Mini Mental State Examination (MMSE) (O’Bryant et al., 2008) to obtain a cognitively healthy sample. From these 754 participants, six had to be excluded due to failed MRI preprocessing.

We log-transformed IL6, CRP, adiponectin and leptin values to ensure normality and regressed age and sex from all predictors prior to PLSC.

### Magnetic Resonance Imaging

Anatomical T1-weighted images were acquired using a 3 Tesla Siemens Verio MRI scanner (Siemens Healthcare, Erlangen, Germany) with a 3D MPRAGE protocol (inversion time, 900 ms; repetition time, 2300 ms; echo time, 2.98 ms; flip angle, 9°; field of view, 256 mm × 240 mm × 176 mm; voxel size, 1 mm × 1 mm × 1 mm).

We performed voxel-based morphometry (VBM) implemented in SPM 12 to obtain voxelwise estimates of GMV. First, a study-specific template was created from 1186 healthy participants of the LIFE-Adult study aged 60 years or older using DARTEL. After non-linear, iterative registration of the white and gray matter segmentations to this template, the resulting flowfields were applied to the gray matter segmentation. Finally, the images were modulated by the amount of spatial distortion and smoothed with a Gaussian kernel of 8 mm FWHM.

As we were interested in the association of GMV, metabolic and anthropometric measures, we aimed to remove the confounding effect of age, sex and total intra-cranial volume (TIV). Therefore, we regressed age, sex and TIV from the GMV using SPM’s implementation of the General Linear Model. To limit the number of voxels included in the analysis, we only included voxels with gray matter probability of 0.3 and larger in the averaged GMV image. The number of voxels included per participant was 295365.

### Statistical Analysis

#### PLSC Analysis

After preprocessing, the anthropometric and metabolic measures were organized in a matrix *X* with dimensions *N*×*p*. The GMV data was stored in a matrix *Y* with dimensions *N*×*v*. Here, *N* is the number of participants, *p* the number of anthropometric and metabolic measures and *v* the number of GMV voxels. The data matrices were columnwise centered and normalized to eliminate influence of variance differences between measures.

PLSC aims to create latent variables (LV) from the two data sets that maximize their pairwise correlation (Krishnan et al., 2011).

$$m\,a\,x\,i\,m\,i\,z\,e\,\left(C\,o\,v\,\left(X\,u,Y\,v\right)\right)=m\,a\,x\,i\,m\,i\,z\,e\,\left(u^{T}\,X\times Y\,v\right)$$

The solution of this maximization problem is obtained by singular value decomposition (SVD). This operation decomposes the *p*×*v* matrix *X*^*T*^*Y* into three matrices *U*(*p*×*R*), *V*(*v*×*R*) and Δ(*R*×*R*) (Abdi, 2007). *R* is the rank of *X*^*T*^*Y*, e.g., maximally *R* pairs of latent variables can be extracted (here *R* = 9).

$$X^{T}\,Y=U\,\Delta \,V^{T}$$

*U* contains the *R* left singular vectors, Δ is a *R*-by-*R* diagnoal matrix containing the *R* singular values and *V* contains the *R* right singular vectors. The singular vectors, or weights, define the latent variables as linear combinations of the original data.

Specifically, *L*_*x*_ = *Xu* are the LV describing the anthropometric and metabolic measures and *L*_*y*_ = *Yv* are the latent variables describing the GMV. The first pair of LV explains the largest possible correlation between the two data sets; the second pair reveals the largest possible correlation under the constraint that the latent variables are uncorrelated to the first pair, and so on. In the following, the weights defining the obesity LV are referred to as metabolic profile and the obesity and GMV LV are called metabolic and brain score, respectively.

#### Statistical Inference

Significance of the resulting decomposition was tested with two approaches: classical permutation-bootstrap inference (Efron and Tibshirani, 1986; McIntosh and Lobaugh, 2004) and a cross-validation framework (Smith et al., 2015).

In the permutation-bootstrap inference, we first determined the significance of the pairs of LV, starting with a full decomposition, e.g., the maximal number of 9 LV pairs. We randomly permuted the rows of the obesity data matrix *X* while leaving the row order of the imaging data matrix *Y* unchanged. This process was repeated 2000 times and for each permutation, the SVD was performed and null-distributions of singular values were built for the pairs of LV. Based on these distributions, a *p*-value was attributed to the original, unpermuted singular values.

$$p=\frac{N\left(permutedsingularvalue>originalsingularvalue\right)}{N_{p\,e\,r\,m\,u\,t\,a\,t\,i\,o\,n\,s}}$$

A pair of LV was considered significant at a level of α = 0.05. The amount of explained covariance was calculated as the singular value of the significant LV pair divided by the sum of all singular values.

When we considered a pair of latent variables generalizable based on the permutation-derived *p*-value, the reliability of the individual weights was estimated by using bootstrap sampling with replacement. We bootstrapped 2000 times from the participants data in the *X* and *Y* matrices and calculated the SVD. Dividing the weights by their standard error derived from bootstrapping yielded a Z-like score, which indicated stable weights when Z > 2.3 (Krishnan et al., 2011).

In order to visualize most stable regions, we performed a cluster-forming procedure using FSL’s cluster-command with an arbitrary threshold of Z > 5.

We also implemented a cross-validation framework according to Smith et al. (2015). First, we randomly selected 80% of the sample for a training set (N∼499) and 20% for a test set (N∼149). Then, we estimated the SVD in the training set and calculated the LV for the test data set by multiplying the resulting weights for brain and obesity-related measures with the raw values of the test data set. This yielded a metabolic and a brain score for each individual. Then, we calculated the correlation of metabolic and brain scores across participants for the test set. In order to establish a null distribution, we randomly permuted the metabolic data matrix within the test set and reprojected the weights derived from the training set onto the permuted raw data (*N* = 1000). Then we compared the correlation of the resulting “random” scores to the original correlation and derived a *p*-value.

We repeated this procedure twenty times, e.g., twenty different training-test datasets, and calculated the average correlation of the true projection, the average correlation of the random projections and the number of significant permutation tests.

The analyses were implemented in python 2.7, based on previously published scripts for PLSC^1^. All code is openly available under https://github.com/fBeyer89/metabolic_ VBM_PLSC.

#### Comparison of BMI and Metabolic Score

We assessed whether a LV based on anthropometric and metabolic measures was a better predictor of GMV than BMI alone. To do so, we compared two models predicting the total GMV, adjusted for intracranial volume, derived by Freesurfer segmentation software, version 5.3.0. Model 1 comprised age, sex and BMI as predictors, and Model 2 additionally included the metabolic score. The model comparison was performed with the function anova in R version 3.2.3.

### Sensitivity Analyses

We performed sensitivity analyses using the permutation-bootstrap approach (9 LV, 2000 permutations, 2000 bootstraps) to assess the effect of different confounding factors.

First, we excluded *N* = 240 participants with IL6 values below the detection threshold (<1.5 pg/ml) to ensure that the skewed distribution arising from this threshold did not affect the result. Then, we repeated the analysis excluding 25 participants with markedly high CRP values (>10 mg/l, Macy et al., 1997). Similarly, we repeated the analysis excluding one participant with an outlier value in adiponectin (<average value – 5σ).

Medication intake, specifically antidiabetic and antihyperlipidemic treatment, might have confounded the laboratory measures of HbA1c and total cholesterol/HDL in our analysis. Therefore, we derived medication-adjusted HbA1c and total cholesterol/HDL values by regressing out the intake of antidiabetic or antihyperlipidemic treatment. A binary definition of antidiabetic treatment was used based on self-reported diagnosis or medication intake. For antihyperlipidemic treatment we only took into account self-reported medication intake. Then, we repeated the permutation-bootstrap analysis (9 LV, 2000 permutations, 2000 bootstraps) with the medication-adjusted HbA1c and total cholesterol/HDL values.

Higher BMI is closely linked to higher blood pressure, which is itself linked to GMV differences (Beauchet et al., 2013). Therefore, we investigated the contribution of systolic blood pressure to the metabolic score. We performed another permutation-bootstrap analysis (10 LV, 2000 permutations, 2000 bootstraps) with the previous metabolic and anthropometric measures and additionally including systolic blood pressure, adjusted for age and sex. This measure was available in *N* = 740 participants.

### Cognitive Function

Cognitive function was assessed with the Consortium to Establish a Registry for Alzheimer’s Disease (CERAD) neuropsychological test battery. We calculated three composite scores for executive function, memory and processing speed according to previous studies (Kharabian Masouleh et al., 2016; Zhang et al., 2018).

Verbal fluency tests “Animals” and “S”-words and the ratio of Trail-making Test (TMT) parts B and A were used to define executive function (Zexecutive = z(number of Animals VF) + z(number of S-words VF) – z(TMT(part B - part A)/part A)/3).

Memory was based on the 10-item CERAD word learning task. The composite score was calculated from the number of learnt words over three consecutive learning trials, the number of correctly recalled words after a delay of about 5 min and the number of correctly recognized word from a list of 20 mixed words [Z memory = (z(number of learned words) + z(number of recalled words) + z(number of recognized words)/3)].

Processing speed was estimated by the inverse value of the time needed to complete TMT part A [Z processing = - z(TMT part A)].

One participant missed data for the TMT.

## Results

### Demographics

See Table 1 for demographic and obesity-related characteristics of the study and Supplementary Figure S7 for bivariate correlations of the anthropometric and metabolic measures used for PLS analysis.

**TABLE 1:** Demographics of the sample.

	**Mean**	**Standard deviation**	**Minimum**	**Maximum**
Age [years]	68.4	4.8	60.00	79.00
Sex [males/females]	416/332 (55.6%/44.4%)			
BMI [kg/m2]	27.7	4.1	16.8	42.3
WHR	0.96	0.08	0.73	1.17
HbA1c [%]	5.53	0.59	3.84	12.38
Diabetes diagnosis or intake of anti-diabetic medication [no/yes]	642/106 (85.8/14.2%)			
Total Cholesterol [mmol/l]	5.86	1.08	2.26	10.76
HDL [mmol/l]	5.85	1.10	1.66	10.76
Anti-hyperlipidemic medication [no/yes]	565/183 (75.5%/24.5%)			
CRP [mg/l]	2.95	7.40	0.16	146.92
IL6 [pg/ml]	3.76	3.88	1.50	64.74
Adiponectin [ng/ml]	7710.4	4662.6	2.0	34744.5
Leptin [ng/ml]	12.187	12.135	0.000	88.290
Systolic blood pressure [mmHg]	134.44	16.24	86.33	195.67

*Given is mean ± standard deviation (min, max). BMI, body mass index; WHR, waist-to-hip ratio; HbA1c, glycated hemoglobin; HDL, high-density lipoprotein; IL6, interleukin-6; CRP, C-reactive protein.*

### Main Analysis

In the main analysis, we included BMI, WHR, HbA1c, total cholesterol, HDL, CRP, IL6, adiponectin and leptin as anthropometric and metabolic measures, and VBM-based GMV as brain morphometric measure.

Based on the permutation-bootstrap approach, the first two pairs of LV were significant (LV1: *p* < 0.001, LV2: *p* = 0.0075) and explained 44.9%/14.6% of the covariance of anthropometric, metabolic and GMV measures, respectively.

**FIGURE 1:**
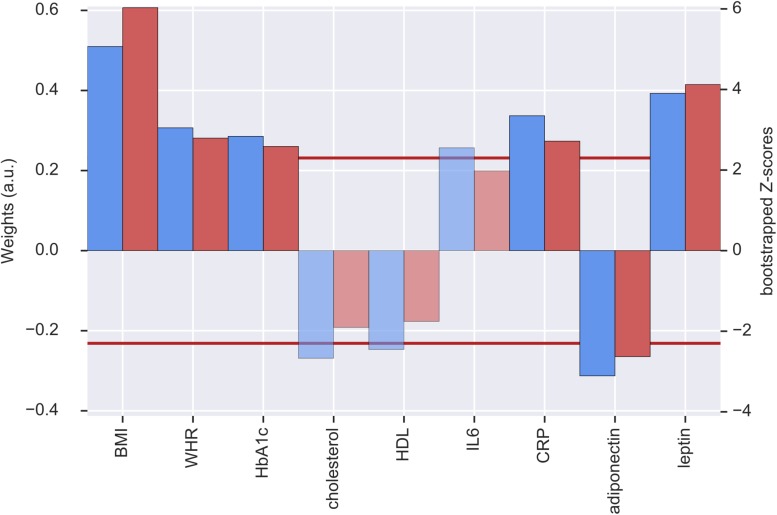
Weights (blue, left *y*-axis) and *Z*-scores (red, right *y*-axis) of the metabolic latent variables (LV) from the first pair of LV. Red line indicates the threshold of bootstrapped *Z*-score = 2.3 All measures with a *Z*-score < 2.3 are shown as transparent. a.u., arbitrary unit; BMI, body mass index; WHR, waist-to-hip ratio; HbA1c, glycated hemoglobin; HDL, high-density lipoprotein; IL6, interleukin-6; CRP, C-reactive protein.

The first pair of LV represents an association of higher metabolic risk and lower GMV in different regions of the brain. The metabolic profile was mainly driven by positive contributions of BMI, WHR, HbA1c, CRP and leptin and a negative contribution of adiponectin. BMI (0.50), leptin (0.39), and CRP (0.33) had the highest weights (see Figure 1). These contributions were stable based on the bootstrapped *Z*-value of *Z* > 2.3.

**FIGURE 2:**
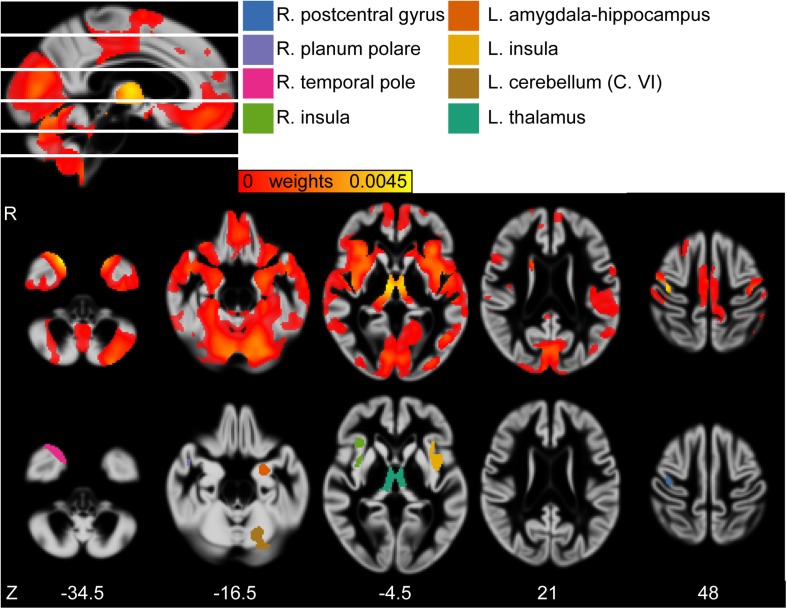
**(First row)** Sagittal view of the gray matter volume (GMV) weight map of first latent variables (LV). White lines indicate axial slices shown in second row. **(Second row)** Axial view of GMV weight map of first LV. **(Third row)** Axial view of clusters derived from bootstrapped *Z* > 5. MNI-coordinates are given in mm for axial orientation (Z). Legend refers to clusters shown in third row. L, left; R, right.

For the GMV, a distributed pattern in temporal, frontal and occipital lobe as well as subcortical regions and cerebellum had reliable negative weights (*Z* > 2.3) (see Figure 2, upper row). Based on an arbitrary threshold of *Z* > 5, thalamus, left cerebellum (Crus VI), bilateral insular cortex, left amygdala/hippocampus, right temporal pole, right planum polare and right postcentral gyrus were identified as most reliable regions (see Table 2 and Figure 2, lower row).

**TABLE 2:** Significant clusters of gray matter volume (GMV) weight map of the first set of latent variables (LV) according to multivariate partial least squares correlation (PLSC) analysis, according to bootstrapped *Z* with an arbitrary threshold of *Z* > 5.

**Region**	**Number of voxels**	**MNI coordinates of peak voxel (X,Y,Z)**	**Bootstrapped *Z* at peak voxel**	**Weight at peak voxel**
Thalamus (Th.)	1408	55, 71, 57	6.94	0.0045
Left cerebellum (Crus VI)	756	68, 40, 40	5.83	0.0037
Left insular cortex	419	83, 84, 50	5.9	0.0031
Left amygdala/hippocampus	353	75, 80, 35	5.66	0.0031
Right insular cortex	347	37, 84, 50	5.53	0.0032
Right temporal pole	343	46, 90, 26	7.27	0.0044
Right planum polare	139	30, 80, 43	5.82	0.0034
Right Postcentral gyrus	101	34, 71, 80	6.3	0.0039

The correlation of the GMV and metabolic LV was *r* = 0.246 (*p* < 0.001, *N* = 748).

**FIGURE 3:**
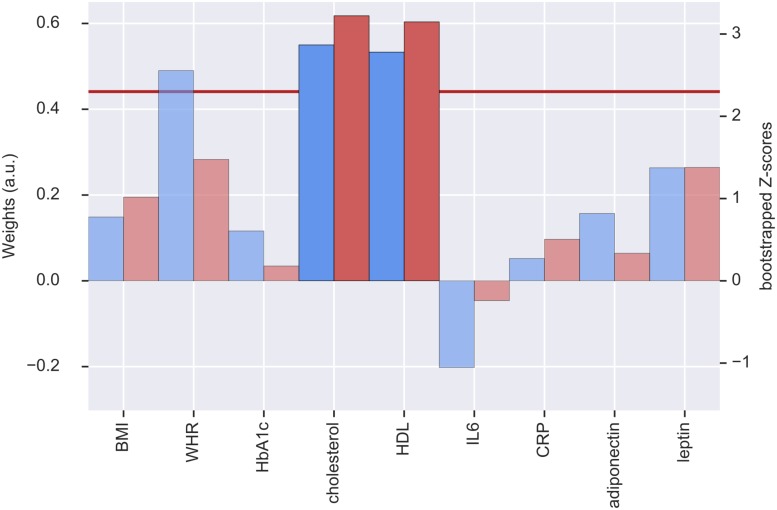
Weights (blue, left *y*-axis) and *Z*-scores (red, right *y*-axis) of the metabolic latent variables (LV) from the second pair of LV. Red line indicates the threshold of bootstrapped *Z*-score = 2.3. All measures with a *Z*-score < 2.3 are shown as transparent. a.u., arbitrary unit; BMI, body mass index; WHR, waist-to-hip ratio; HbA1c, glycated hemoglobin; HDL, high-density lipoprotein; IL6, interleukin-6; CRP, C-reactive protein.

**FIGURE 4:**
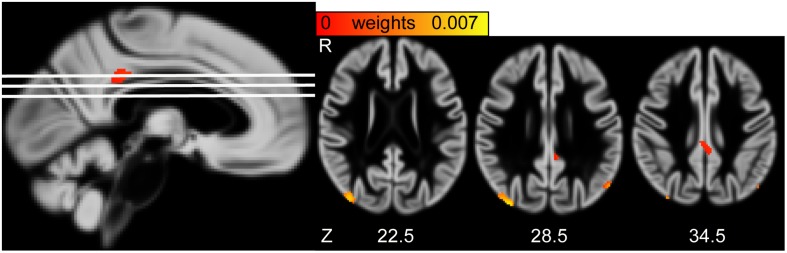
**(Left)** Sagittal view of the gray matter volume (GMV) saliency weight map of second latent variables (LV). White lines indicate axial slices shown on the right. **(Right)** Axial view of GMV weight map of second LV. MNI-coordinates are given in mm for axial orientation (Z). R, Right.

The second pair of LV had positive and reliable weights for total cholesterol and HDL (see Figure 3). Three clusters of reliable, positive weights were found in the posterior cingulate and bilateral lateral occipital cortex (see Figure 4). The correlation of the second pair of latent variables was *r* = 0.22 (*p* < 0.001, *N* = 748).

We applied a cross-validation framework to assess the reliability of the first two pairs of LV.

The mean and standard deviation of the correlation between the first LV in the test data sets was 0.241 ± 0.074 (*N* = 149). Out of 20 training-test data sets, the first LV did not reach nominal significance of *p* < 0.05 in two data sets where *p*-values were 0.13 and 0.094.

The mean and standard deviation of the correlation between the second LV in the test data sets was 0.10 ± 0.07 (*N* = 149). Out of 20 training-test data sets, the second LV did not reach nominal significance of *p* < 0.05 in thirteen data sets.

While in the permutation-bootstrap approach the amount of variance explained in the original dataset was significant, the correlation of the second pair of LV was not stable in the cross-validation approach. In PLSC, the second pair of LV is constrained by its orthogonality to the first pair. This means that the second pair of LV represented an orthogonal mode of variation within the anthropometric and metabolic measures that might, given the relative low amount of variance explained (∼15%), have a limited interpretation. For the following analysis, we thus focus on the results for the first pair of LV.

#### Comparison of BMI and Metabolic Score

In Model 1, age, sex and BMI were all significant predictors of total GMV adjusted for TIV (see Table 3). Yet, model 2 which additionally included the metabolic score, predicted total GMV slightly better (see Table 3, model comparison: *F* = 4.1, *p* < 0.042). This indicates metabolic measures explain additional variance compared to anthropometry when investigating GMV differences related to higher BMI.

**TABLE 3:** Statistics according to linear regression models predicting total gray matter volume (GMV).

**Total GMV adjusted for head size**	**Model 1 (*R*^2^_adj_ = 0.207)**			**Model 2 (*R*^2^_adj_ = 0.213)**		
		
	**st. β**	***T***	***p***	**st. β**	***T***	***p***
Age	–0.27	–8.3	<0.0001	–0.27	–8.3	<0.0001
Sex	0.30	9.3	<0.0001	0.31	9.4	<0.0001
BMI	–0.17	–5.4	<0.0001	–0.09	–1.73	0.084
Metabolic score (LV1)				0.11	2.1	0.042

*Model 1 included age, sex, and body mass index (BMI). Model 2 additionally included the metabolic score from the first set of latent variables (LV) according to multivariate partial least squares analysis (see text for details). st. β, standardized ß-coefficient of the linear regression; R^2^_*adj*_, adjusted R^2^.*

### Sensitivity Analysis

We performed sensitivity analysis to detect possible confounding effects on the first pair of LV.

#### IL6 Below Detection Threshold

When excluding participants with IL6 values below the detection threshold, the first pair of LV was similar to the main analysis (*p* < 0.001, explained covariance = 0.41). BMI and leptin had negative weights. Adiponectin had a stable positive contribution as well as HDL and cholesterol which reached the threshold of *Z* > 2.3 in this analysis (see Supplementary Figure S1). WHR and CRP did not contribute reliably to the metabolic score of the first LV. The weights of the GMV score remained essentially unchanged.

#### Outliers in CRP and Adiponectin

Here, we excluded participants with markedly high CRP values (>10 mg/l, *N* = 25) who might have had an acute infection or another reason for elevated CRP at the time of the assessment. The first pair of LV were very similar to the main analysis, except that the bootstrapped Z-value of CRP dropped to 2.26 below the pre-defined threshold of 2.3 (see Supplementary Figure S2). The pattern of the GMV score was essentially unchanged.

When removing one participant with an outlying value in adiponectin (*N* = 1), we did not see any differences in the metabolic and GMV scores (see Supplementary Figure S3).

#### Systolic Blood Pressure as Additional Predictor

We added systolic blood pressure as another important cardiovascular risk factor to see whether it explained additional variance in the obesity-GMV association. There was no reliable contribution of systolic blood pressure to the first obesity LV (see Supplementary Figure S4) but the positive contribution of total cholesterol and HDL to the metabolic score became significant.

The GMV score did not change.

#### Analysis Adjusting for Intake of Antidiabetic and Antihyperlipidemic Medication

To see whether the observed mode of covariation was driven by manifest metabolic disease, like diabetes or hyperlipidemia, we regressed the treatment of those conditions from the respective variables HbA1c and total cholesterol/HDL. After this correction, HbA1c did not contribute to the first obesity LV anymore, indicating that the heightened levels of HbA1c in diabetic patients might have driven the involvement of HbA1c in the first obesity LV (see Supplementary Figure S5). The weights of the GMV LV were unchanged.

### Association of the Metabolic Score and Cognitive Function

We performed linear regression to determine the association of the brain and metabolic scores and three sum scores of cognitive function.

**FIGURE 5:**
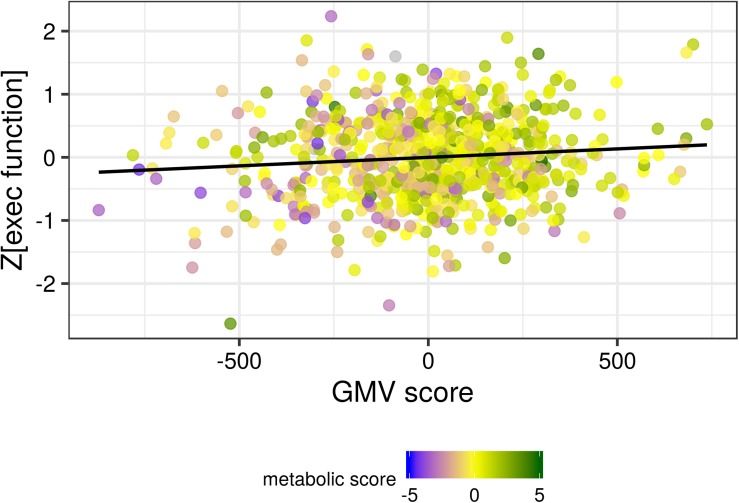
The gray matter volume (GMV) score was positively associated with the sum score of executive function. A higher metabolic score (corresponding to lower BMI and indicated by the color coding shown in the legend) was associated with both higher GMV score and higher executive function. Black line indicates the locally smoothed average.

For executive function, we found a significant positive association of brain and metabolic score with the sum score (standardized β_ob_ = 0.084, *p* = 0.021; standardized β_brain_ = 0.098, *p* = 0.007). Higher score on the brain LV and higher score in the metabolic LV (with negative loadings of BMI and WHR) both predicted better executive function (see Figure 5). When excluding one participant with outlying value in adiponectin, the pattern was even more pronounced (see Supplementary Figure S6).

No association between brain or metabolic LV was found for the memory sum score.

Processing speed was positively associated with the brain LV but not the metabolic LV (standardized β_ob_ = −0.019, *p* = 0.59; standardized β_brain_ = 0.132, *p* < 0.001).

## Discussion

In this study, we showed that a metabolic profile of obesity predicted lower GMV in a large population-based sample of older adults. Higher BMI, WHR, leptin, HbA1c, CRP and lower adiponectin levels were jointly associated with reduced GMV in cortical, subcortical and cerebellar brain regions, including the thalamus, insular cortex and temporal pole. We used two inference schemes and performed sensitivity analysis for potential confounding of outliers, detection thresholds and medication intake. Higher scores in the metabolic score and the GMV score predicted better executive function performance.

### The Metabolic Profile

The metabolic profile of the first LV represents common metabolic dysregulations in obesity (Van Gaal et al., 2006).

Leptin and BMI had the highest weights in the metabolic profile. BMI is the ratio of body weight to height and an indirect estimate of body fat (Frankenfield et al., 2001). Yet, due to age-related changes in body composition, BMI might lack sensitivity to detect individuals with excess body fat in the older population (Romero-Corral et al., 2008; Stenholm et al., 2008). Here, leptin levels are a more accurate estimator of total body fat, as they increase with the amount of overall adipose tissue in the body (Considine et al., 1996; Ostlund et al., 1996).

In the present sample, the high weights of leptin and BMI in the profile indicate that the overall amount of adipose tissue is a strong predictor of reduced GMV in older adults. This suggests that factors underlying a sustained energy imbalance, like sedentary lifestyle or intake of energy-dense food, might be important determinants of gray matter structure in aging (Erickson et al., 2010; Dingess et al., 2017; Kreutzer et al., 2017).

Considering the central effects of leptin, it is also possible that leptin levels are directly associated with brain structure. Yet, previous studies showed mixed results in that both lower and higher leptin levels predicted reduced GMV in older individuals (Narita et al., 2009; Rajagopalan et al., 2013). To reconcile these contradictory findings it is important to consider the BMI distribution of the study population. Obesity goes along with elevated leptin levels and leptin resistance, which may lead to central deficiency and impaired beneficial action of this hormone in the brain (Paz-Filho et al., 2010). Therefore, both low and chronically elevated leptin levels might be associated with structural brain differences, especially in the hippocampus (Lieb et al., 2009; Witte et al., 2016).

In line with the literature, adiponectin was inversely related to leptin and BMI in the metabolic profile of LV1 (Havel, 2002). As adiponectin might have positive effects on brain function related to its insulin-sensitizing and anti-inflammatory properties (Lihn et al., 2005), reduced levels of adiponectin might indirectly contribute to reduced GMV.

Smaller weights were found for WHR and CRP in the metabolic profile. WHR – in contrast to BMI and leptin – reflects the distribution of adipose tissue and is considered a measure of visceral adiposity. Having a higher amount of visceral adipose tissue is linked to a higher risk for cardiovascular disease (Lee et al., 2008) due to specific functions of this fat tissue. Visceral fat tissue releases pro-inflammatory cytokines, like IL6, and short-chain fatty acids, factors involved in the development of the metabolic syndrome and arteriosclerosis (Després and Lemieux, 2006; Bergman Richard et al., 2012; Item and Konrad, 2012). In line with these findings, the inflammation marker CRP contributed to the metabolic profile, even though the association is attenuated when excluding participants with extremely elevated CRP-levels. This points to the low specificity of CRP which is a measure of both localized and systemic inflammation and might therefore be confounded by participants with acute infections in the sample.

IL6, a pro-inflammatory cytokine secreted by visceral adipose tissue (Fontana et al., 2007), had no significant weight in the metabolic profile although it added to the profile in a similar direction as CRP. This might be due to the reduced sensitivity of the laboratory assessment and the resulting skewed distribution. When excluding participants with IL6 values below the detection threshold, CRP no longer contributed to the metabolic profile. This indicates that in participants with IL6 values below the detection threshold, relevant variance regarding inflammatory processes is captured in the high-sensitivity CRP we assessed.

Overall, the contribution of WHR and CRP to the metabolic profile shows that beyond increased whole-body fat mass (measured by BMI and leptin), visceral adipose tissue and related systemic inflammation play a role in obesity-related GMV reductions. Pro-inflammatory cytokines can cross the blood-brain barrier (Hsuchou et al., 2012) and thereby promote inflammatory reactions in the central nervous system (Erickson et al., 2012; Spielman et al., 2014). One example is the chronic activation of microglia, that triggers the production of reactive oxygen species (ROS) and pro-inflammatory cytokines, and may lead to neuronal loss (Spielman et al., 2014; Colonna and Butovsky, 2017).

Long-term glucose marker HbA1C had a significant and reliable weight in the metabolic profile, indicating that disturbed glucose and insulin metabolism is another pathway linking obesity to reduced GMV. High blood glucose levels enchain the production of advanced glycation end-products (AGEs) which trigger the production of ROS and may lead to inflammatory reactions (Yan et al., 2008). Thereby, elevated glucose levels might damage vasculature or enhance neuroinflammation (Yan et al., 2008). Insulin resistance may also damage the brain, given the importance of insulin for neuromodulatory and –protective processes as well as memory and cognition (Craft and Watson, 2004; Blázquez et al., 2014).

Our sample included around 100 individuals with diabetes and when we adjusted for intake of antidiabetic medication, HbA1c no longer contributed to the metabolic profile. This result indicates that the contribution of HbA1c might have been driven by GMV difference in diabetic patients. In line with this interpretation, pronounced GMV differences have been reported in diabetic patients while more subtle associations, mostly limited to the hippocampus, have been reported in the range of normal glucose metabolism (Benedict et al., 2012; Moran et al., 2013; Shaw et al., 2017).

Regarding the lipid metabolism, we did not find a stable contribution of total or HDL cholesterol to the metabolic profile in the main analysis. Still, we found that total and HDL cholesterol positively covaried with the metabolic profile, this effect was more pronounced when we excluded participants with IL6 below the detection threshold or included systolic blood pressure. In the literature, no or negative associations have been reported for total cholesterol and GMV (Enzinger et al., 2005; Chen et al., 2006; Walhovd et al., 2014) while one study found a positive association of HDL and GMV (Ward et al., 2010). Adjusting for intake of lipid-lowering medication did not change the result. Interestingly, in our sample, total cholesterol and HDL were highly correlated, and against our expectations, total cholesterol was negatively correlated with BMI (standardized β = −0.13, *p* < 0.001, adjusted for age and sex). This might explain why the lipid measures were not reliably included into the profile.

Chronically elevated blood pressure is commonly found in older age and strongly associated with obesity. Elevated blood pressure is a strong predictor of brain damage, in form of lacunar infarcts, white matter hyperintensities and GMV loss (Beauchet et al., 2013; Suzuki et al., 2017; Haight et al., 2018). Still, systolic blood pressure did not contribute to the metabolic profile when it was included along with the other predictors. We noticed that BMI was weakly negatively associated with systolic blood pressure in this sample (standardized β = −0.075, *p* = 0.04, adjusted for age and sex) which might explain why systolic blood pressure was not included into the profile.

### The GMV Pattern Associated With the Metabolic Profile

We found a consistent association of the metabolic profile and lower GMV in thalamus, bilateral insular cortex, left amygdala-hippocampus, temporal pole and the cerebellum. These findings are in line with the literature where mostly negative associations between obesity and GMV are reported (Willette and Kapogiannis, 2015). More specifically, a recent meta-analysis reported BMI-associated reductions of GMV in temporal pole and cerebellum (Garcia-Garcia et al., 2018). The cerebellum not only contributes to the planning of motor actions but also plays an important role for cognition (Buckner et al., 2011). Importantly, atrophy patterns related to neurodegenerative disease reflect cerebellar-cortical connectivity patterns, e.g., the cerebellar regions which are functionally connected to the default mode network show atrophy in Alzheimer’s disease (Guo et al., 2016). It is therefore plausible that obesity-associated metabolic factors, associated with differences in specific brain networks, might also contribute to cerebellar atrophy (Haight et al., 2015; Beyer et al., 2017; Kharabian Masouleh et al., 2018).

Differences in thalamic, insula and amygdalar-hippocampal GMV have not been reported in the meta-analysis, but were found in univariate analysis of obesity-related factors such as CRP (Corlier et al., 2018), HbA1c (Reitz et al., 2016), and BMI (Kharabian Masouleh et al., 2016). The current study investigated the univariate association of BMI and GMV in a partly overlapping sample with Kharabian Masouleh et al. (2016) (*N* = 412 or 55% overlap with the present sample) and found similar clusters in thalamus, parahippocampal gyrus and temporal lobe. These regions show a decline in GMV over the adult life span, and possibly, obesity and related metabolic factors enhance this effect, as proposed by the increased brain age observed in white matter of obese participants (Storsve et al., 2014; He et al., 2015; Ronan et al., 2016).

In contrast to previous studies, the metabolic profile was not predominantly associated with frontal GMV in our analysis. Still, medial orbitofrontal and superior frontal cortex were reliably (*Z* > 2.3) linked to the metabolic profile. Studies suggested that reduced GMV in obesity might not only be a consequence but also a potential genetic risk factor for developing obesity (Opel et al., 2017). Thus, genetic factors, among others, might have contributed to the observed GMV differences in our study. Possible mediators include executive functions and impulsive behavior which might impact eating behavior and thereby lead to weight gain (Chuang et al., 2015). However, as our analysis did not include genetic or behavioral traits, in addition to its cross-sectional design, interpretation of causes and consequences underlying GMV differences is limited.

Expanding the study by Kharabian Masouleh et al. (2016), we used a multivariate strategy to characterize the association of obesity and GMV in older adults. Accordingly, the individual metabolic profile score explained more variance in total GMV than BMI alone. This analysis was independent of the actual pattern of GMV associated with the metabolic score. Yet, the overall amount of variance in total GMV explained by the metabolic score is relatively small (∼3%) compared to the variance explained by age and sex (∼18%).

### Cognitive Function

Regarding the relevance of our findings for cognitive function, exploratory analyses suggested that executive function was gradually decreased along the axis of the first obesity-brain LV. Both lower metabolic LV and higher GMV LV were associated with increased performance in the domain of executive function. This result expands previous findings of reduced executive function related to increased BMI reported in a partly overlapping sample by Kharabian Masouleh et al. (2016), and shows that reduced GMV in distributed brain regions might mediate this effect.

We did not find an association of memory performance and the GMV pattern of the first LV. While there was no direct association of BMI and memory performance, (Kharabian Masouleh et al., 2016) found an indirect effect, mediated by GMV in frontal and thalamic clusters. Our analysis was possibly not suited to replicate this region-specific association between brain and cognition, given that we derived a wide-spread GMV pattern which, among others, included frontal and thalamic clusters.

We found a positive association of the GMV pattern and processing speed, but not obesity-related LV and processing speed. This might reflect the fact that both executive function and processing speed sum scores are derived from the trail-making-test, and therefore are partly collinear.

These exploratory results are largely in line with the literature, where mid-life obesity has been linked to reduced cognitive function in various cognitive domains (van den Berg et al., 2009; Prickett et al., 2015). More specifically, our results support the view that executive function might be more affected by vascular risk factors than other cognitive domains, such as verbal memory (Wolf et al., 2007; Debette and Markus, 2010) and that metabolic disturbances linearly add to obesity-related cognitive decline (Singh-Manoux et al., 2012).

### Strengths and Limitations of the Current Study

Strengths of this study include a large, well-characterized participants sample and a comprehensive multivariate analysis employing two validation schemes. Additionally, we performed sensitivity analysis and assessed cognitive function of the participants with a standardized neuropsychological test battery.

The main limitation of this study is that we cannot draw causal inferences based on our cross-sectional data.

We reported a relatively specific metabolic profile and a widespread pattern of GMV loss. Yet, due to our multivariate approach we cannot conclude whether certain metabolic factors, e.g., pro-inflammatory cytokines, mediate the association or if they have independent effects on GMV loss. Furthermore, we cannot test regionally specific associations of single metabolic factors with GMV loss. This problem might be partly overcome by using sparse PLSC techniques in future studies (Monteiro et al., 2016). Introducing a sparsity constraint to the PLSC decomposition reduces the number of features, e.g., voxels, and forces many features to have zero weights. This may aid interpretability especially for high-dimensional MRI data. Another drawback of the PLSC approach was the limited interpretability of higher-order latent variables which are constrained to be orthogonal to previous LV (Krishnan et al., 2011).

## Conclusion

Taken together, we provided evidence that a metabolic obesity profile characterized by increased body fat, visceral adiposity and systemic inflammation was associated with a widespread pattern of decreased GMV. The brain-obesity covariation was stable in two validation schemes and predicted executive function in a large sample of older adults without diagnosis of cognitive impairment. We suggest that this unfavorable metabolic profile might contribute to reduced executive function via damage to the gray matter in widespread brain regions.

Following our study, further research is needed to establish the causal relationship between obesity, decreased gray matter volume and cognitive function in aging. Our results indicated a main contribution of overall fat mass and visceral adiposity, which should be tested in longitudinal studies. Given the importance of lifestyle factors in mid-life for cognitive function later in life, these studies might benefit from considering body weight trajectories or using cumulative measures of metabolic burden such as “obesity pack years” (Abdullah et al., 2011; Pedditizi et al., 2016).

Furthermore, investigating measures of brain health beyond gray matter structure, such as imaging markers of cerebral small vessel disease and white matter microstructure, might help to understand the complete picture linking obesity, cardiovascular risk and cognitive decline in aging.

## Data Availability

The datasets generated for this study are available on request to the corresponding author.

## Ethics Statement

This study was carried out in accordance with the Declaration of Helsinki and approved by the institutional ethics board of the Medical Faculty of the University of Leipzig. All subjects gave written informed consent.

## Author Contributions

FB developed the research question, performed the analysis, created the figures, and drafted the manuscript. SM refined the research question, helped with the analysis, and revised the manuscript. JK conducted the biomarker analysis and revised the manuscript. MS designed the study and revised the manuscript. SR designed the study, implemented the cognitive testing, and revised the manuscript. SR-H and AV designed the study and revised the manuscript. AW refined the research question, discussed the data analysis, and drafted and revised the manuscript.

## Conflict of Interest Statement

The authors declare that the research was conducted in the absence of any commercial or financial relationships that could be construed as a potential conflict of interest.

## Footnotes

1 https://libraries.io/github/chrisfilo/pypls
