# Supplementary material for: A Metabolic Obesity Profile Is Associated With Decreased Gray Matter Volume in Cognitively Healthy Older Adults
Source: Front Aging Neurosci. 2019 Aug 2;11:202. doi: 10.3389/fnagi.2019.00202 (PMC6688742; doi:10.3389/fnagi.2019.00202)
Supplement: Supplementary file 1 [file Data_Sheet_1.docx]

**Supplementary File**

Sensitivity analysis:

IL6 below detection threshold
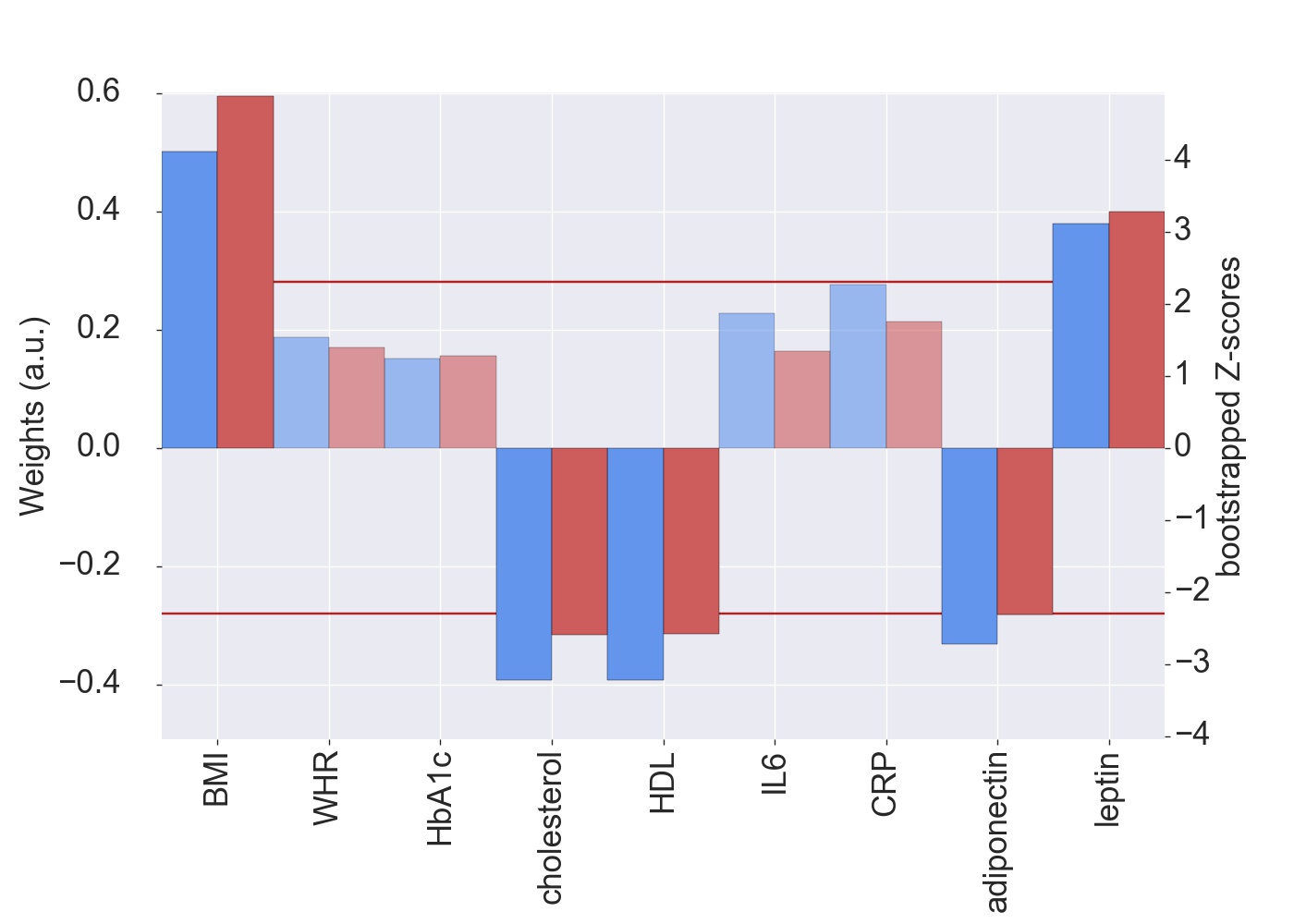


Supplementary Figure S1: Weights (blue, left y-axis) and Z-scores (red, right y-axis) of the anthropometric and metabolic measures for the first pair of LV, shown for the IL6 detection threshold sample (N=508). All measures with a Z-score < 2.3 are shown as transparent. a.u., arbitrary unit; BMI, body mass index; WHR, waist-to-hip ratio; HbA1c, glycated hemoglobin; HDL, high-density lipoprotein; IL6, interleukin-6; CRP, C-reactive protein

Outliers in CRP and adiponectin


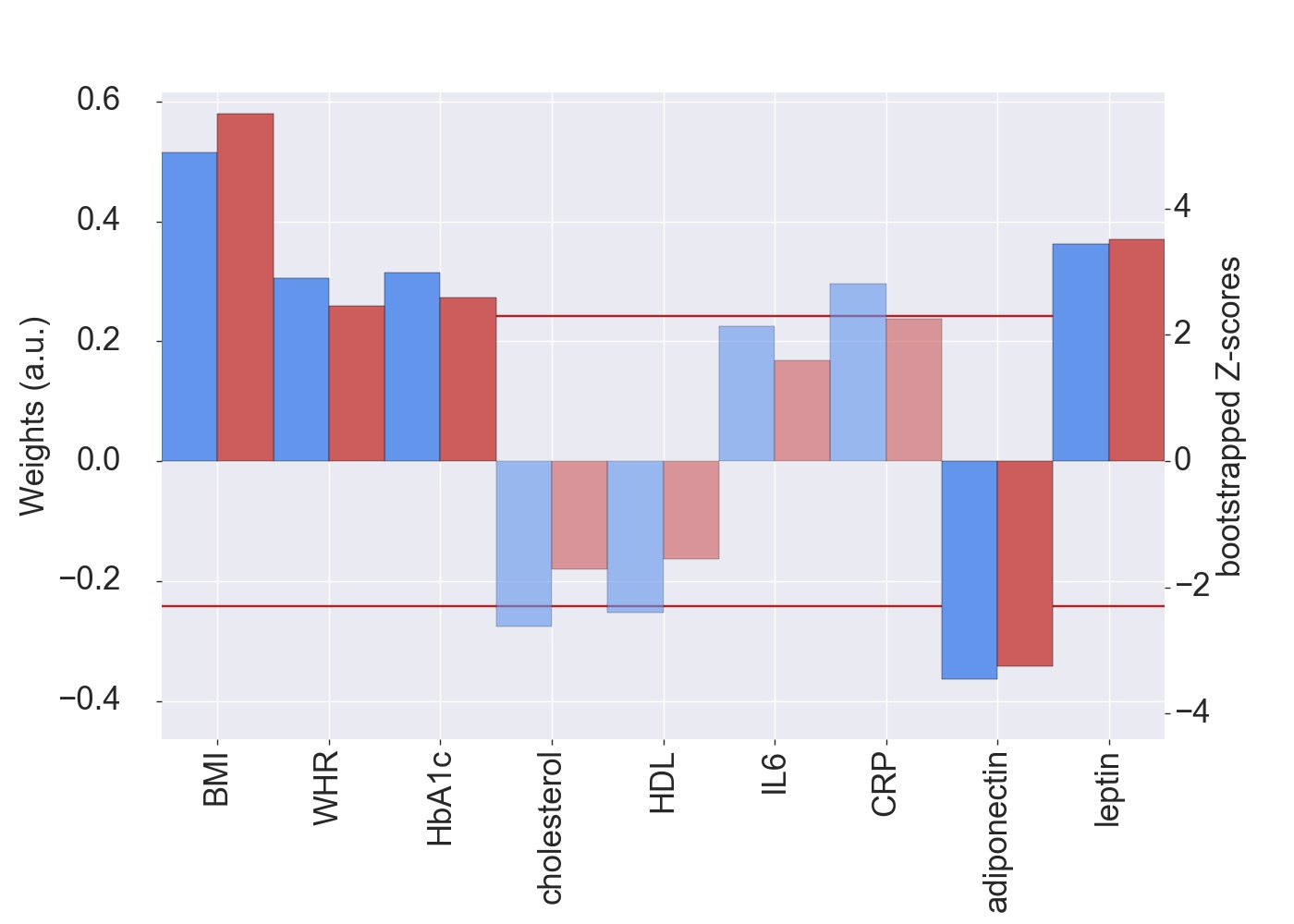


Supplementary Figure S2: Weights (blue, left y-axis) and Z-scores (red, right y-axis) of the anthropometric and metabolic measures for the first pair of LV for the sample without participants with outlying CRP values (N=723). All measures with a Z-score < 2.3 are shown as transparent. a.u., arbitrary unit; BMI, body mass index; WHR, waist-to-hip ratio; HbA1c, glycated hemoglobin; HDL, high-density lipoprotein; IL6, interleukin-6; CRP, C-reactive protein


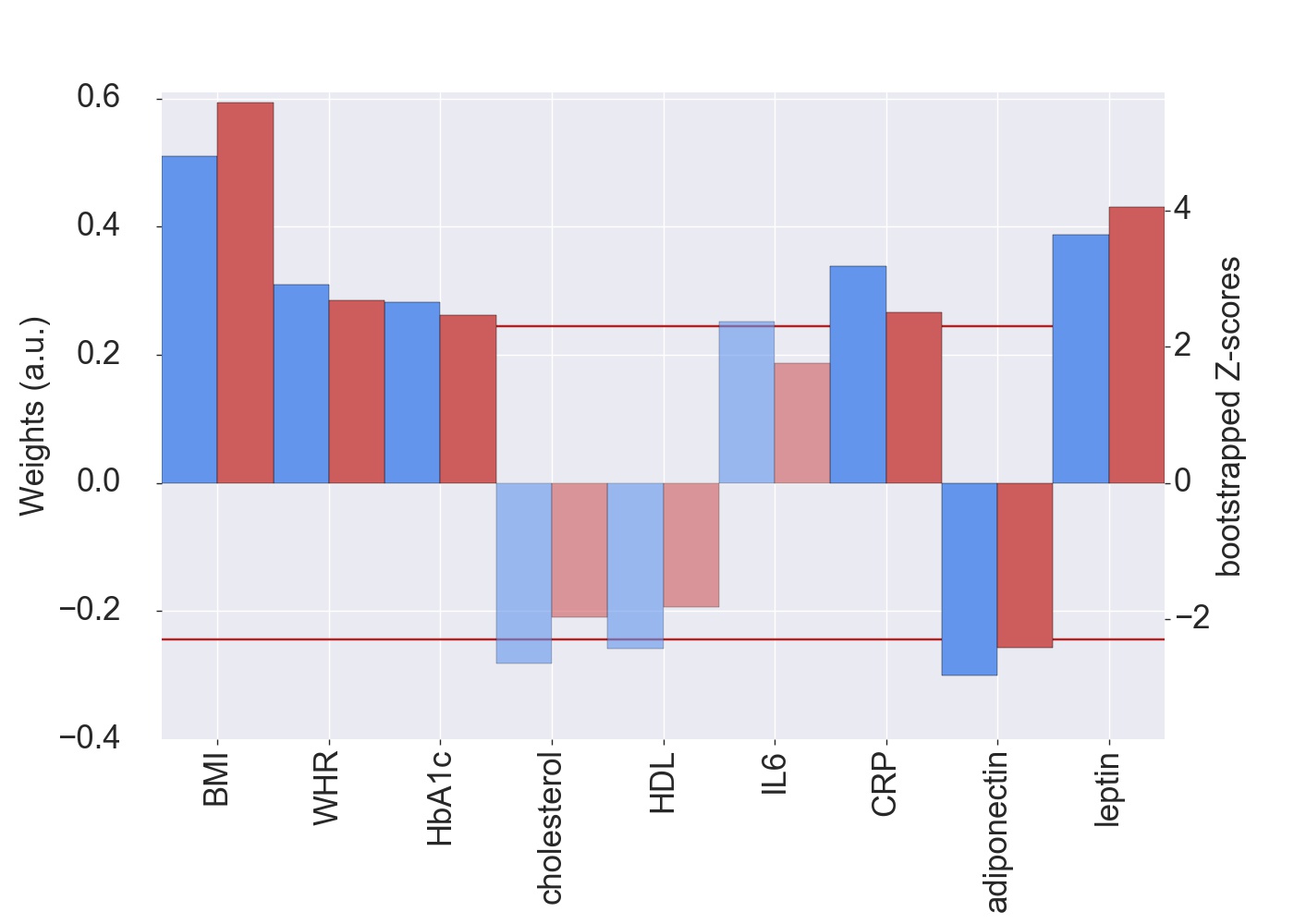


*Supplementary Figure S3: Weights (blue, left y-axis) and Z-scores (red, right y-axis) of the anthropometric and metabolic measures for the first pair of LV for the sample without participant with outlying adiponectin value(N=747). All measures with a Z-score < 2.3 are shown as transparent. a.u., arbitrary unit; BMI, body mass index; WHR, waist-to-hip ratio; HbA1c, glycated hemoglobin; HDL, high-density lipoprotein; IL6, interleukin-6; CRP, C-reactive protein*

Systolic blood pressure as additional predictor


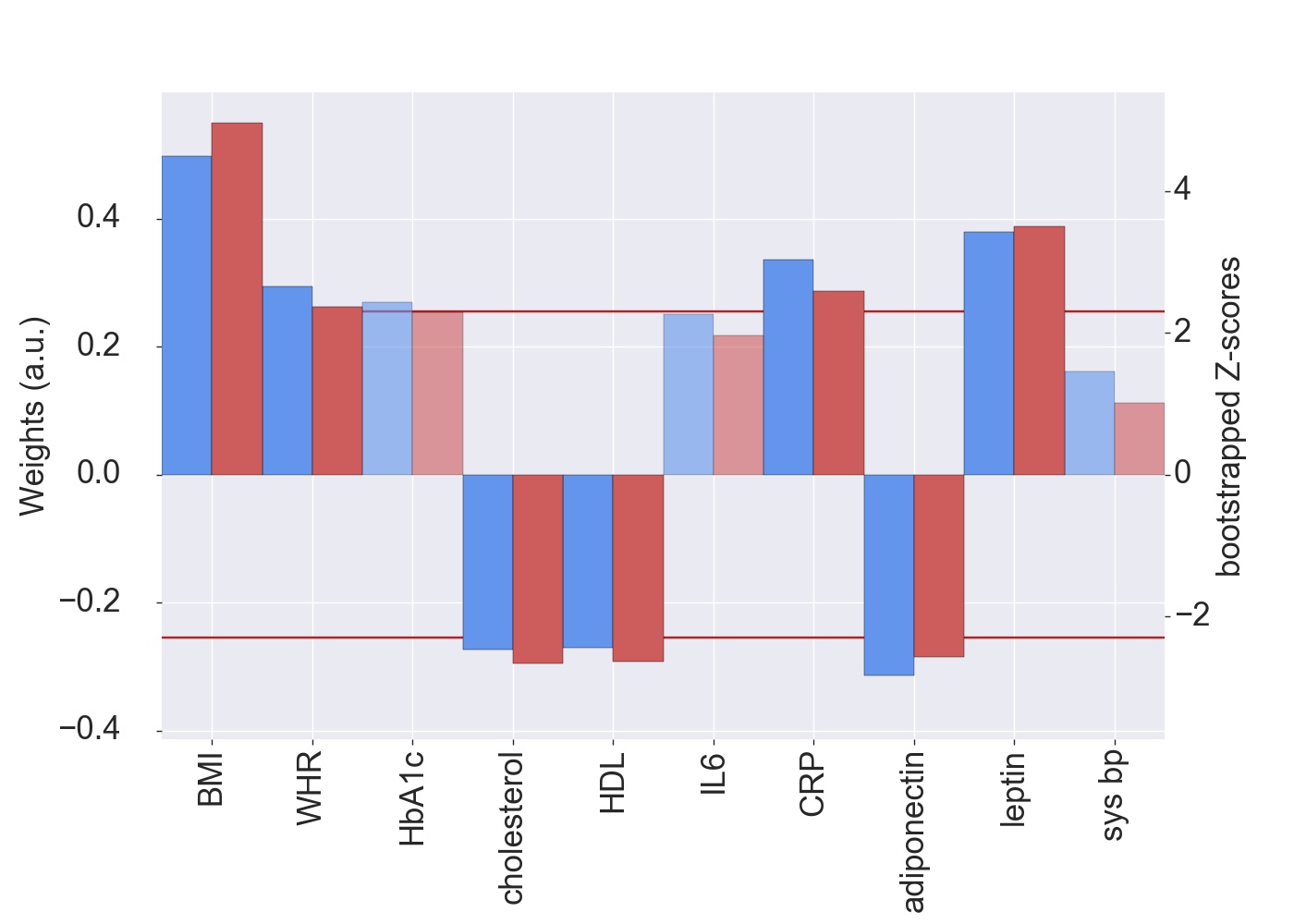


*Supplementary Figure S4: Weights (blue, left y-axis) and Z-scores (red, right y-axis) of the anthropometric and metabolic measures for the first pair of LV including systolic blood pressure (sys bp). All measures with a Z-score < 2.3 are shown as transparent. a.u., arbitrary unit; BMI, body mass index; WHR, waist-to-hip ratio; HbA1c, glycated hemoglobin; HDL, high-density lipoprotein; IL6, interleukin-6; CRP, C-reactive protein*

Analysis adjusting for intake of antidiabetic and antihyperlipidemic medication


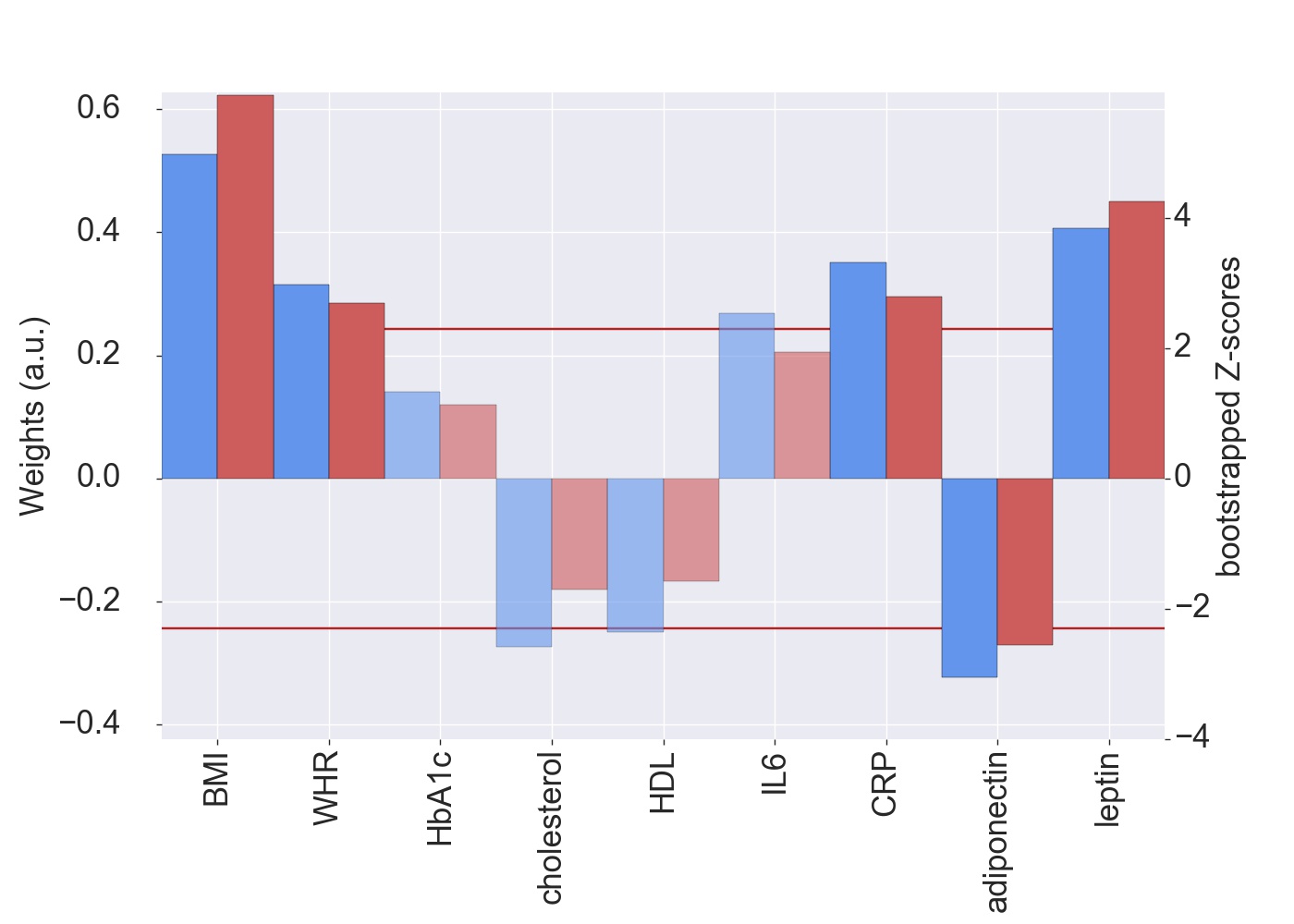


*Supplementary Figure S5: Weights (blue, left y-axis) and Z-scores (red, right y-axis) of the anthropometric and metabolic measures for the first pair of LV after adjusting for intake of antidiabetic and antihyperlipidemic medication. All measures with a Z-score < 2.3 are shown as transparent. a.u., arbitrary unit; BMI, body mass index; WHR, waist-to-hip ratio; HbA1c, glycated hemoglobin; HDL, high-density lipoprotein; IL6, interleukin-6; CRP, C-reactive protein*

Association of the metabolic score and cognitive function (without outlier in adiponectin)


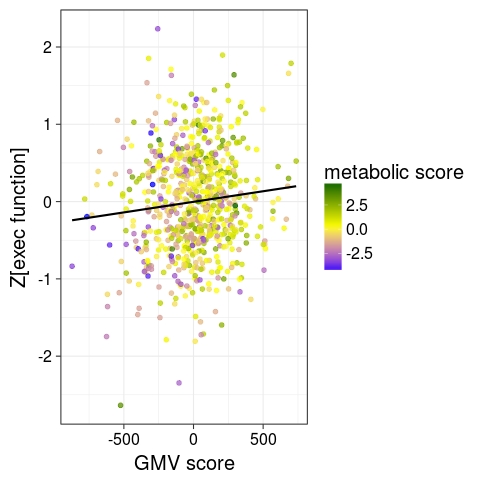


*Supplementary Figure S6: LV brain scores are positively associated with the sum score of executive function. Higher scores in obesity-related LV is associated with both higher score in brain LV and higher executive function. This sample excluded one participant with outlying values in adiponectin (N=746).*

**Pairwise correlations of anthropometric and metabolic markers**

*
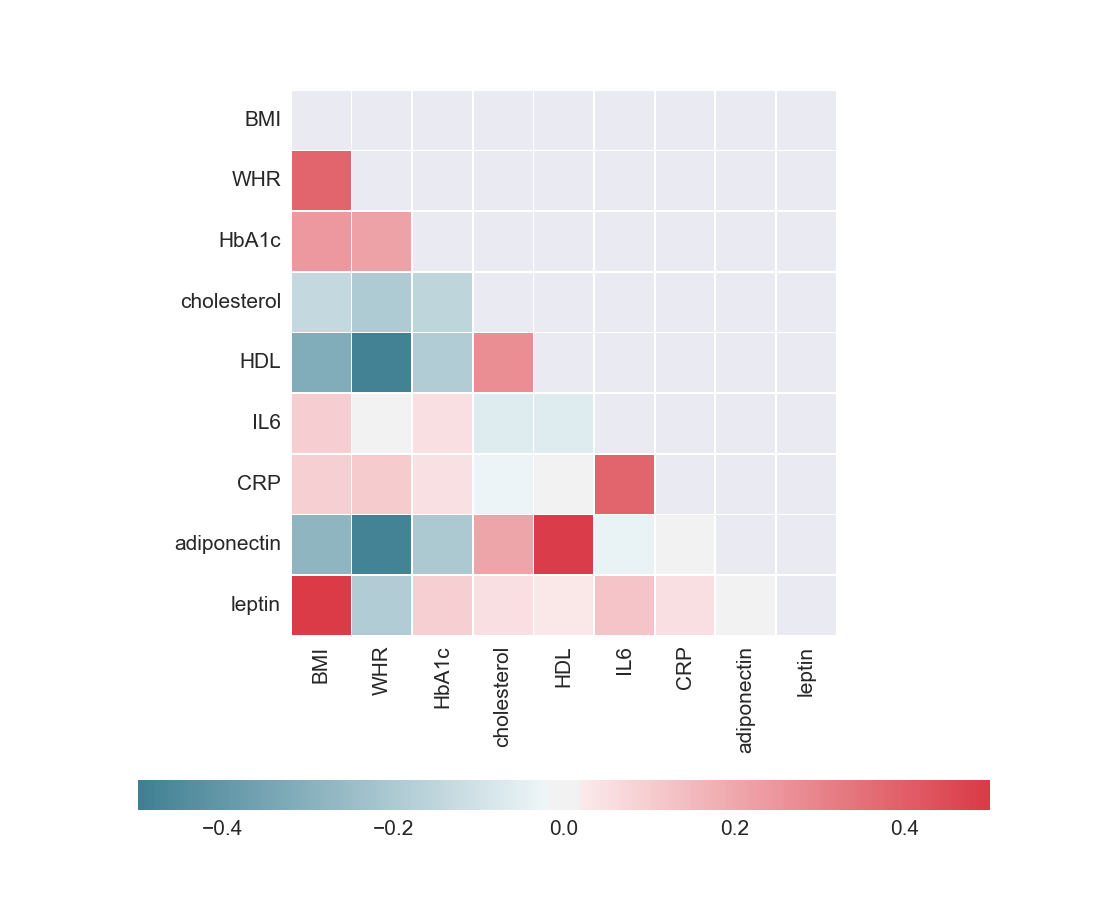
 Supplementary Figure S7: Pairwise correlations of anthropometric and metabolic measures used for partial least squares analysis. The color scale indicates negative correlations in blue and positive correlations in red.*
